# Frontal Lobe Hemodynamic Responses to Painful Stimulation: A Potential Brain Marker of Nociception

**DOI:** 10.1371/journal.pone.0165226

**Published:** 2016-11-02

**Authors:** Christopher M. Aasted, Meryem A. Yücel, Sarah C. Steele, Ke Peng, David A. Boas, Lino Becerra, David Borsook

**Affiliations:** 1 Center for Pain and the Brain, Harvard Medical School; Boston, Massachusetts, United States of America; 2 Department of Anesthesiology, Perioperative and Pain Medicine, Boston Children’s Hospital and Harvard Medical School; Boston, Massachusetts, United States of America; 3 Martinos Center for Biomedical Imaging, Department of Radiology, Massachusetts General Hospital and Harvard Medical School; Boston, Massachusetts, United States of America; Université catholique de Louvain, BELGIUM

## Abstract

The purpose of this study was to use functional near-infrared spectroscopy (fNIRS) to examine patterns of both activation and deactivation that occur in the frontal lobe in response to noxious stimuli. The frontal lobe was selected because it has been shown to be activated by noxious stimuli in functional magnetic resonance imaging studies. The brain region is located behind the forehead which is devoid of hair, providing a relative ease of placement for fNIRS probes on this area of the head. Based on functional magnetic resonance imaging studies showing blood-oxygenation-level dependent changes in the frontal lobes, we evaluated functional near-infrared spectroscopy measures in response to two levels of electrical pain in awake, healthy human subjects (n = 10; male = 10). Each subject underwent two recording sessions separated by a 30-minute resting period. Data collected from 7 subjects were analyzed, containing a total of 38/36 low/high intensity pain stimuli for the first recording session and 27/31 pain stimuli for the second session. Our results show that there is a robust and significant deactivation in sections of the frontal cortices. Further development and definition of the specificity and sensitivity of the approach may provide an objective measure of nociceptive activity in the brain that can be easily applied in the surgical setting.

## Introduction

While subjective measures of pain may be helpful in the clinic, there are a number of clinical conditions where subjects are either drowsy or unconscious during tissue damage, such as surgery. Providing adequate anesthesia during surgery is routine, but we do not have a good measure of nociceptive stimuli that may act on brain systems. Our prior report suggests that during anesthesia, activation of certain brain regions may take place during nociceptive stimulation[1]. Frontal lobe activation during nociceptive stimulus has been reported by others. [2–4]. Here we wished to extend these findings using functional near infrared spectroscopy (fNIRS) with more complete coverage of the frontal lobe in order to determine an optimal frontal area in which such signals are most easily defined. As summarized in our prior report[1], frontal lobe activity is involved in nociceptive signaling. This has implications for placement of probes with regard to signal detection and ease of placement in conditions such as evaluation of pain in clinical conditions (e.g., under anesthesia). We also have previously reported activation in the somatosensory cortex to nociceptive stimuli, confirming the nature of the parallel observations in the frontal lobe for the same stimulus using fNIRS [5].

fNIRS provides a non-invasive approach for the study of cortical cerebral hemodynamic fluctuations by passing two harmless wavelengths of light, such as 690 nm and 830 nm[5], through the scalp, cerebral spinal fluid, and cortical volumes. As this light travels, it is altered by oxygenated and deoxygenated hemoglobin, which are among the dominant absorbers of near-infrared light in biological tissue [6]. The backscattered light is detected by photodiodes and through the application of the modified Beer-Lambert Law it is possible to calculate changes in cerebral concentrations of deoxygenated (HbR) and oxygenated (HbO) hemoglobin, as well as total hemoglobin concentration (HbT) [1].

The experience of pain involves a number of brain regions, several of which are located in the cortical regions observable with fNIRS. The rationale for evaluating the frontal lobes was also considered in light of multiple functional magnetic resonance imaging (fMRI) studies of pain or nociception reporting activation in the frontopolar prefrontal cortex, including Brodamann Area 10. [7, 8–12]. Decreased activation to noxious painful stimuli has been observed in such nociceptive evoked pain studies (viz., [11]). Furthermore, our previous work indicated that a frontal lobe cortical response could be used to differentiate between low and high pain intensity stimuli, as we were able to do over the somatosensory cortex[5]. Placement of fNIRS sensors on the forehead would make the application of the technology easy to use in clinical situations (e.g., sedation or anesthesia). The objective of the present study was to evaluate responses to a noxious stimulus and determine if (1) specific regional activation using fNIRS over two polarfrontal cortical (medial and lateral) regions for the same noxious stimulus rated for two levels of pain intensity (low and high); and (2) whether there are any differences between the responses to noxious stimuli in these two regions.

## Methods

The study was approved by the Institutional Review Board (IRB) of the Massachusetts General Hospital and met the scientific and ethical guidelines for human pain research of the Helsinki Accord and the International Association for the Study of Pain.

### NIRS Probe and System

**Fig 1:**
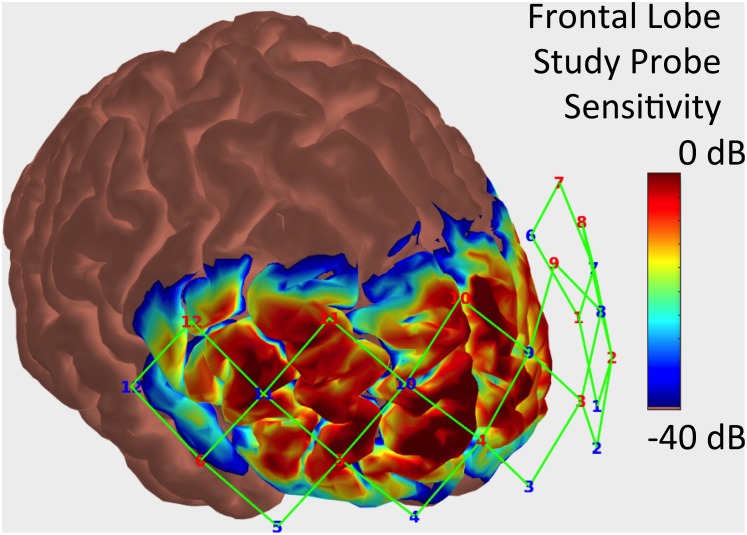
Sensitivity profile and sensor channel locations for probe used in this study. The sensitivity of each probe to detecting brain hemodynamics is represented on a logarithmic color scale ranging from 0 dB (red) to -40 dB (blue) times the maximum sensitivity. The source locations (red numbers), detector locations (blue numbers), and channels (green lines) are further detailed in Fig 2.

Cerebral hemodynamic activity was recorded using a multichannel functional near-infrared spectrometer operating at 690 and 830 nanometer wavelengths (TechEn Inc. MA, USA, CW7 System). The probe contained 12 sources, 12 standard separation detectors, and 12 short separation detectors. Fig 1 illustrates the layout of the sources and standard separation detectors. Standard separation detectors were positioned 30 millimeters from adjacent sources and short separation detectors were located 8 millimeters from a single source.

### Study protocol and subject population

Ten healthy subjects were included in the study (right handed, male, mean age 24.4 ± 5.8 years). Each subject gave informed written consent prior to the experiments. Subjects with a history of neurological trauma or psychiatric disorders, or who were unable to remain still for 6 consecutive minutes, were excluded.

### Pain Stimuli and Ratings

Prior to performing the experiment, a 5 Hz electrical stimulation was applied to each subject’s left thumb (Neurometer CPT, Neurotron, Baltimore, MD). For subjective measures of pain, we used the verbal rating scale—verbal analogue scale[13], which has been shown to be a reliable scale[14] as measure of pain intensity. Details of the pain ratings were explained to the subjects prior to the stimuli being applied: “on an 11 point scale where 0 = no pain and 10 = maximal pain please define the stimulus that produces a 3/10 pain level and in a separate rating a 7/10 pain level to an increasing electrical stimulus”. Thus, we evaluated two levels of pain intensity: low (3/10) and high (7/10) intensities in each individual. The levels of stimulus for each rating in each individual were then used during the NIRS recordings. During the experiment, semi-randomized sequences of the low and high pain stimuli, lasting 5 seconds in duration, separated by 25-second periods of rest, were applied in two 6-minute sets, separated by a 30-minute resting period. This resulted in a total of 12 low intensity (3/10) and 12 high intensity (7/10) noxious stimuli in a sequence, with equal numbers of each per 3-minute section, for use in the analysis.

### Data Processing Stream

**Fig 2:**
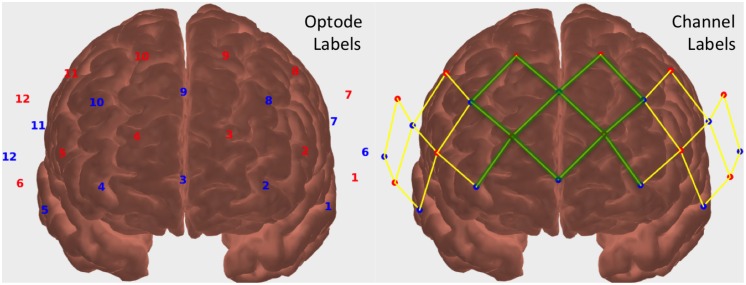
(left) Probe source (red) and detector (blue) labels and (right) channels allocated into the ‘central’ (green) and ‘lateral’ (yellow) groups for analysis.

Analysis was carried out using the open source software Homer2, which is implemented in Matlab (Mathworks, Natick, MA), and the process described in Yücel et al., 2015[5]. The processing stream parameters that were used are detailed in Table 1. The sensor channels were split into two groups to evaluate whether central or lateral regions respond more to nociceptive stimulus, as depicted in Fig 2. The ‘central’ section was composed of the 12 source-detector pairs listed in Table 2 and predominately covered the superior frontal gyri, while the ‘lateral’ results included the remaining 8 source-detector pairs on each side, for a total of 16 channels (Table 3), predominately covering middle and inferior frontal gyri. Some overlap occurs because the probe channels were selected independent of their cortical projections. Each of the 6-minute runs was split to separately analyze the first and second three minutes to assess whether a habituation effect occurs.

**Table 1:** Parameters used for Homer2 processing stream. Please see Homer2 documentation for further information on function parameters (http://homer-fnirs.org/tutorials/).

Prune Channels	dRange: [1e4, 1e7] SNR: 2 SDrange: [0, 45] reset: 0
Motion Artifact by Channel	tMotion: 0.5 tMask: 1 STDEVthresh: 50 AMPthresh: 5
Stim Rejection	tRange: [-5, 20]
Bandpass Filter	HPF: 0 LPF: 0.5
OD2Conc	PPF: [6, 6]
DeconvHRF_DriftSS	tRange: [-2, 20] glmSolveMethod: 1 idxBasis: 1 paramsBasis: [1, 1] rhoSH_ssThresh: 15 flagSSmethod: 1 driftOrder: 3 flagMotionCorrect: 0

Key: Prune Channels dRange = Acceptable Range of Raw Data Values, SNR = Signal-to-Noise Ratio, SDrange = Source-Detector Separation Range to Pass, tMotion = Motion Artifact Motion Parameter, tMask = Motion Artifact Mask Parameter, STDEVthresh = Motion Artifact Threshold for Standard Deviation, AMPthresh = Motion Artifact Threshold for Amplitude, Stim Rejection tRange = Time Range Used to Reject Stimulus Marks Near Motion Artifacts, Bandpass Filter HPF = High Pass Filter (in Hz), LPF = Low Pass Filter (in Hz), OD2Conc = Optical Density to Concentration, PPF = Partial Pathlength Factor, DeconvHRF_DriftSS = Deconvolution Hemodynamic Response Function with Drift and Short Separation, tRange = Time Range for Hemodynamic Response Function, glmSolveMethod = General Linear Model Solve Method, idxBasis = Basis Function Used for the Hemodynamic Response Function, paramsBasis = Parameters Used for the Basis Function, rhoSH_ssThresh = Maximum Threshold for Short Separation Channel Spacing, flagSSmethod = Flag Short Separation Method Used, driftOrder = Drift Order Used for Regression Polynomial, flagMotionCorrect = Flag Motion Correction.

**Table 2:** Source and detector pairs from Fig 2 and the Montreal Neurological Institute (MNI) coordinates and segmentation labels for the center point of each optode pair.

Source	Detector	MNI Coordinates (mm)	Segmentation Label (AAL)
3	2	-14 52 5	left superior frontal gyrus, medial
3	3	-6 73 12	left superior frontal gyrus, medial
3	8	-21 67 26	left superior frontal gyrus, dorsolateral
3	9	-5 64 25	left superior frontal gyrus, medial
4	3	12 75 14	right superior frontal gyrus, medial
4	4	22 59 8	right superior frontal gyrus, dorsolateral
4	9	11 69 28	right superior frontal gyrus, medial
4	10	26 65 26	right middle frontal gyrus
9	8	-16 53 35	left middle frontal gyrus
9	9	-2 43 26	left superior frontal gyrus, medial
10	9	8 49 30	right superior frontal gyrus, medial
10	10	24 53 35	right superior frontal gyrus, dorsolateral

**Table 3:** MNI coordinates and segmentation labels for ‘lateral’ probe channels in the results.

Source	Detector	MNI Coordinates (mm)	Segmentation Label (AAL)
1	1	-42 40 3	left middle frontal gyrus
1	6	-61 14 12	left inferior frontal gyrus, opercular part
1	7	-45 28 19	left inferior frontal gyrus, triangular part
2	1	-38 48 4	left middle frontal gyrus
2	2	-29 56 4	left middle frontal gyrus
2	7	-46 46 17	left middle frontal gyrus
2	8	-38 61 22	left middle frontal gyrus
5	4	38 58 4	right middle frontal gyrus, orbital part
5	5	54 54 2	right middle frontal gyrus
5	10	36 53 18	right middle frontal gyrus
5	11	56 45 18	right inferior frontal gyrus, triangular part
6	5	62 42–2	right inferior frontal gyrus, orbital part
6	11	44 26 17	right inferior frontal gyrus, triangular part
6	12	59 12 9	right inferior frontal gyrus, opercular part
7	6	-48 9 24	left precentral gyrus
7	7	-41 17 25	left inferior frontal gyrus, triangular part
8	7	-41 31 27	left inferior frontal gyrus, triangular part
8	8	-32 50 37	left middle frontal gyrus
11	10	32 44 34	right middle frontal gyrus
11	11	42 26 23	right inferior frontal gyrus, triangular part
12	11	57 22 28	right inferior frontal gyrus, opercular part
12	12	64 10 25	right precentral gyrus

### Statistics

Statistical analysis was performed using MATLAB to perform paired t-tests to determine whether there was a significant difference between the two stimulus intensities across subjects for each test segment. Statistical significance was assessed with a p-value of 0.05 corrected for multiple comparisons (p<0.0125) across medial and lateral averaged source-detector pair signals and across 2 time points.

## Results

### Frontal Lobe Responses

**Fig 3:**
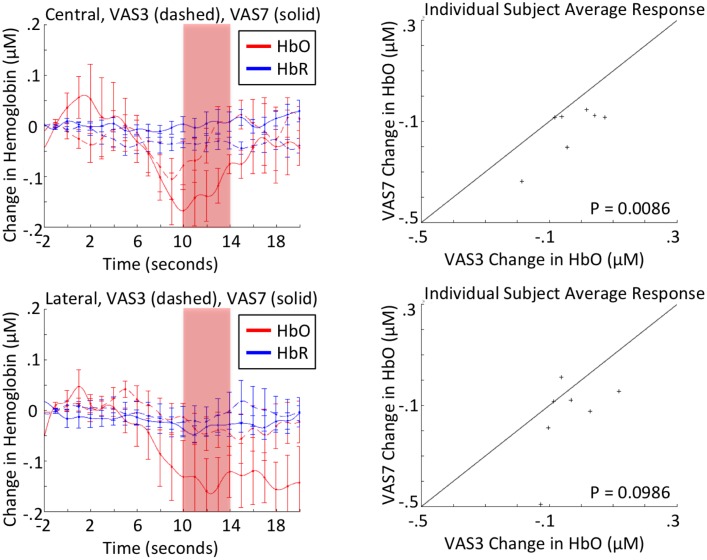
NIRS responses to low and high pain intensity stimuli. *Top Left*: The figure shows the group average hemodynamic response observed from central frontal channels, predominantly positioned over the superior frontal cortices. *Bottom Left*: Hemodynamic response observed from lateral channels, predominantly positioned over the bilateral medial frontal cortices. Oxygenated (red) and deoxygenated hemoglobin (blue) are depicted for the response to high (7/10; solid line) and low (3/10; dashed line) stimuli with standard error bars. *Right top and bottom*: The mean oxygenated hemoglobin response for each subject over the period 10–14 seconds post-stimulus, observed from central frontal channels (top) and from lateral channels (bottom). The number of stimuli in the analysis for each subject can be found in Table 4. The black line is a visual aid with a slope of one and zero y-intercept. A verbal analogue scale (VAS) was used to determine pain levels corresponding to a 3/10 (low pain intensity) and 7/10 (high pain intensity).

Using the automated signal-to-noise ratio and artifact detection algorithms in Homer2, the data from three subjects were identified as not meeting the minimum criteria for signal quality based on the criteria in Table 1 and were excluded from the group analysis. In most cases of this type, exclusion occurs as the result of weak signal strength or channel saturation (raw detector signal values under 1e4 or over 1e7) or excessive subject motion (standard deviation of raw detector signal greater than 50 or amplitude change greater than 5 over a 0.5-second interval). The remaining seven data sets contained a total of 65 usable low pain intensity stimuli and 67 high pain intensity stimuli (see Table 4), and were then analyzed to determine the group mean hemodynamic response over the central and lateral probe sections presented in Fig 3. The pattern of hemodynamic activity across the central channels during the first three stimuli of each type for the first run produced the hemodynamic response that best replicated the results obtained in Yücel et al., 2015[5]. Averaging the change in oxygenated hemoglobin across the time interval 10 to 14 seconds post-stimulus produces a statistically significant differentiation between the two stimuli (i.e., VAS 3/10 vs. VAS 7/10).

**Table 4:** Number of stimuli included in the analysis.

Subject	Run 1 (six minutes)				Run 2 (six minutes)				Subtotal	
	1st Three Minutes		2nd Three Minutes		1st Three Minutes		2nd Three Minutes			
	VAS 3/10	VAS 7/10	VAS 3/10	VAS 7/10	VAS 3/10	VAS 7/10	VAS 3/10	VAS 7/10	VAS 3/10	VAS 7/10
1	3	3	3	3	3	3	2	2	11	11
2	3	3	1	2	1	2	2	2	7	9
3	2	2	3	3	2	3	2	3	9	11
4	3	1	2	3	2	2	2	3	9	9
5	3	3	3	2	1	1	2	2	9	8
6	3	3	3	3	1	1	2	3	9	10
7	3	3	3	2	3	2	2	2	11	9
Subtotal	20	18	18	18	13	14	14	17	65	67

### Changes in Frontal Response Over Time

To further investigate the effect of habituation on the brain’s response to noxious stimuli, the same process that was used to distinguish high pain intensity from low pain intensity stimuli during the first three stimuli of each type, was applied to each of the four time segments, separately analyzing the central and lateral regions. From this analysis it was determined that there was a distinguishable difference between the two stimulation intensities over the central channels for both the first and second sets of stimuli during the first 6-minute session, but that the difference lost significance during the second 6-minute period of stimuli (Table 5). No statistically significant difference was found between the HbO response for the lateral channels during any of the time segments (Table 6).

**Table 5:** Evaluating the significance of the difference in the response across the central channels during the period 10–14 seconds post-stimulus for the VAS7 and VAS3 electrical stimuli.

Evaluating VAS7 vs. VAS3 for Central Channels	p-Value of paired t-test (n = 7)
Run 1, 1st Three Minutes	0.0086
Run 1, 2nd Three Minutes	0.0469
Run 2, 1st Three Minutes	0.1373
Run 2, 2nd Three Minutes	0.1544

**Table 6:** Evaluating the significance of the difference in the response across the lateral channels (average of both sides) during the period 10–14 seconds post-stimulus for the VAS7 and VAS3 electrical stimuli.

Evaluating VAS7 vs. VAS3 for Lateral Channels	p-Value of paired t-test (n = 7)
Run 1, 1st Three Minutes	0.0986
Run 1, 2nd Three Minutes	0.6472
Run 2, 1st Three Minutes	0.2835
Run 2, 2nd Three Minutes	0.4978

## Discussion

In this study we demonstrated that the frontal lobe metric is robust and reproducible and we determined that the signal of interest is most strongly present in the centrally positioned optodes. Specifically, we determined that the relative change in oxygenated hemoglobin concentration over the superior frontal cortices sampled by the central channels is correlated with the perception of pain from electrical nerve stimulation.

### Activation of the Frontal Lobes

As shown in the methods and results (see Fig 1), measures of the fNIRS signals captured with the central channels cover the surface of the superior frontal gyrus. Prior PET and fMRI studies have reported activation in the superior frontal lobe in experimental pain studies[15]. The function of the region includes non-pain processing, including working memory[16], and its role in pain has been established from a number of studies, for example, subdural electrophysiological measures using evoked painful stimuli[17] and fMRI studies on pain [18]. Of these studies, the electrophysiological study is perhaps the most salient as it comes closest to the approach we have taken here. The function in pain/nociceptive processing remains unknown but putative processes include cognitive evaluating prediction errors for aversive stimuli[19]. In this study we observed a decrease in activity correlated with noxious stimulation as well as a reduction in this effect that may be attributed to stimulus salience. Periodontal pain stimulation has been reported to produce a similar decrease in deoxygenated hemoglobin concentration in the frontal cortices [3]. However, studies utilizing a cold pressor test or mechanical force to induce pain have indicated the hemodynamic response may be specific to the type of nociceptive stimulus [4, 20]. Differences between the electrical stimuli that produced low and high pain reports were significant for the central channels but fell short of significance in the laterally placed channels situated on the forehead. Furthermore, the use of short separation channels enhanced the signal compared to using standard separation channels alone. Specifically, using the short separation channels for noise regression substantially improved the ability to distinguish the brain response to noxious stimuli versus autonomic signals in the skin[21].

Given that the frontal regions are involved in a number of processes (viz., multimodal threats and salient stimuli), it is possible that the response we observe may not be specific to nociception. The main frontal region evaluated included the polarfrontal prefrontal cortex or Brodmann Area 10 (see Caveats). Brodman area 10 (BA10) located in the anterior medial frontal cortex. The region is located inferior to Brodmann 9 (superior frontal cortex) and Brodmann 11 (the anterior extension of oribitofrontal cortex). BA 10, also known as the anterior prefrontal cortex, frontopolar prefrontal cortex or rostral prefrontal cortex, is an association cortex is involved in a wide variety of functions including risk and decision making [22], odor evaluation, reward and conflict/threats [23, 24], pain [10], and working memory [25, 26]. While arousal is probably not involved in the response, or only partially contributory in the awake patients and even in the sedated patients, it is unlikely to be so in the fully anesthetized patients (see Caveats below). Furthermore, concomitant with frontal lobe deactivation we observe a parallel increased activation in the primary somatosensory cortex[5, 27].

### Temporal Evaluation of Responses

While we expected to see no differences between the stimulation periods, we observed a diminished response to pain during the second stimulus period, following the 30-minute period of rest. The ability to distinguish the two intensity stimuli in the frontal cortex persists past the first three minutes, unlike the response observed in the somatosensory cortex in earlier studies, where the signal diminished during the second three minutes due to a habituation effect when using the same stimuli[5]. The neurological processes that lead to this decreased response from repeated exposure are well known and include enhanced modulation via frontal-periaqueductal connections [28].

### Benefits of Data acquisition from Forehead

In the clinical settings measures over the frontal area important have substantial advantages over a somatosensory-only monitoring system. Potentially the most significant of these is ease of access. fNIRS requires a reliable interface between the optical equipment and the subject’s scalp, which is complicated by the presence of hair. When hair is present it may require extensive adjustment of the equipment in order to create a reliable optical connection. This could limit widespread use of the technology in medical applications. A second advantage is redundancy. Because measurements from biological processes are notoriously noisy and are often prone to artifacts and unexpected behaviors, by monitoring two brain regions instead of one, when possible, fNIRS has the potential to self-verify and reduce the prevalence of false-positives.

### Caveats

We wish to point out a number of caveats related to the study. (1) Specificity of Response: We do not know that the response is specific to pain. Since we did not utilize non-nociceptive control stimuli, it is not possible to exclude that the observed differences in the responses could be related to differences in stimulus intensity and/or salience for the frontal response in this study. However, while we have reported a similar frontal lobe response to noxious stimuli under anesthesia (see conclusions), the issue of salience during these states is still not well understood. It has been reported that there is a breakdown of functional connectivity during anesthesia but not sensory connectivity[29]. We are unaware of electrophysiological measures of neurons in the frontal lobe regions evaluated in this study. Having said this, it is clear that further evaluation of the experimental process to determine the contribution of salient processing in this paradigm is necessary. Furthermore, additional features may contribute to the observed signal including arousal (salience) or anxiety. (2) Potential Variability of Measures in a Clinical Setting: While we have reported on the use of NIRS in measuring pain under anesthesia[30] or sedation[1], there are still issues related to the measures of evoked pain that need clarification including: 2.1. Habituation of a pain response; 2.2. Differences in evoked vs. ongoing pain; 2.3. The depth of anesthesia or use of opioid analgesics; 2.4. Consistency of measures including signal quality in individual patients/subjects. (3) The need for further Studies: This is a preliminary study and further studies are needed to control for highly salient non-nociceptive stimuli. (4) Specificity of Brain Region: Connectivity based parcellation of human frontal polar cortex has previously been described [31]. We did not define the specific area, but our medial probes covered the region and the lateral probes may have included frontal regions just outside the brain area.

## Conclusion

While we currently interpreted these results as a primary response in the frontal lobe region to a noxious stimuli, it may be a secondary response affecting other networks (e.g., Salience Network[32]). Clearly further evaluation of the sensitivity and specificity of this measure, if successful, may provide a potential marker for evoked nociceptive activity in humans.

## Supporting Information

S1 Dataset Subject HRF’s Run 1 Part 1. (CSV)

S2 Dataset Subject HRF’s Run 1 Part 2. (CSV)

S3 Dataset Subject HRF’s Run 2 Part 1. (CSV)

S4 Dataset Subject HRF’s Run 2 Part 2. (CSV)
